# Presentation of the 1^st^ International Symposium on Strength & Conditioning (ISSC 2011)

**DOI:** 10.2478/v10078-011-0051-8

**Published:** 2011-10-04

**Authors:** Mário C. Marques, Adam Zajac

**Affiliations:** 1 Research Centre for Sport Sciences, Health and Human Development (CIDESD), Vila Real, Portugal; 2 Department of Sport Sciences, University of Beira Interior (UBI), Covilhã, Portugal; 3 Department of Sports Training, Academy of Physical Education, Katowice, Poland

The ISSC 2011 was the first of a series of future events that we hope will help to spread Strength & Conditioning research among Europe. Unfortunately, there is no European association specifically designed to address that same mission. Contrarily, in the USA the National Strength & Conditioning Association (NSCA) is a well-known organization that has been promoting Strength & Conditioning research quality for the last two decades, though its implementation in Europe may be viewed as scarce. Nevertheless, the amount of European sports scientists that perform high-quality research related to strength training and physical conditioning is noticeable. Within this framework, a group of young researchers that work in four public universities (two in Portugal and two in Brazil) decided to implement this symposium and asked for the support of NSCA and, specially, for the support of Dr. Steven Fleck. Indeed, Dr. Fleck provided the necessary “brain” support to build the idea and the NSCA provided the necessary institutional support to make ii possible to happen. Subsequently, the Research Centre for Sports, Health & Human Development (CIDESD), currently hosted in the University of Trás-os-Montes and Alto Douro took full responsibility to welcome the 1^st^ ISSC.

The CIDESD) is a cross-institutional technical and scientific multi-disciplinary unity of applied and fundamental research. This center appears from the network integration, by a consortium agreement of several previous research units originally. The main scope of CIDESD is to promote research on Sports Sciences field, health-related physical activity promotion, in accordance to different stages of human development valuing the relationships between physical activity and sports, health promotion by the influence of human and organizational resources.

The event was held the 15–16^th^ July 2011 in the University of Trás-os-Montes & Alto Douro, at Vila Real (Portugal) and it was mainly addressed to Master and Doctoral students of various Portuguese and European Universities. A total of 9 scientific conferences were presented by 9 expert researchers from 4 different countries (USA, Portugal, Brazil and Spain). Additionally, Dr. Fleck presented also the NSCA mission and its future in Europe and around the World. This was a first small step towards an event that we expect to be able to grow slowly but steadily. In the future, we hope to increase the number of presentations and also to include a typical scientific session open for the research community (oral and or poster presentations).

Another deliver from the ISSC 2011 was the consensus among some of the researchers that participated in the event, to promote a specific research unit dedicated to Strength & Conditioning related issues. This unit will include approximately 20 researchers from the abovementioned 4 countries and it will be initially hosted in CIDESD. Here, the Journal of Human Kinetics (JHK) was also an important partner in this enterprise, since its Editor provided an open space for the scientific diffusion of the event, which is the current supplement of JHK.

